# *Babesia banethi* sp. nov. in red foxes

**DOI:** 10.1186/s13071-025-07179-y

**Published:** 2025-12-11

**Authors:** Domenico Otranto, Mariaelisa Carbonara, Antonio Camarda, Lucas Cafferati Beltrame, Antonio Parisi, Jan Šlapeta

**Affiliations:** 1 Department of Veterinary Medicine, University of Bari, Valenzano, Italy; 2 Department of Veterinary Clinical Sciences, City University of Hong Kong, Hong Kong, China; 3 Istituto Zooprofilattico Sperimentale Della Puglia E Della Basilicata, Foggia, Italy; 4 Sydney School of Veterinary Science, Faculty of Science, The University of Sydney, Camperdown, NSW Australia; 5 Sydney Infectious Diseases Institute, The University of Sydney, Camperdown, NSW Australia

**Keywords:** Babesiosis, *Babesia banethi sp. nov.*, *Babesia vulpes*, Southern Italy, *Ixodes kaiseri*, Red fox

## Abstract

**Background:**

*Babesia* spp. are widespread tick-borne intraerythrocytic protozoa, infecting a broad range of vertebrate hosts. Red foxes are reservoirs of *Babesia vulpes,* belonging to the *Babesia microti*-like group (clade I), and play an important role in the epidemiology of canine and wildlife babesiosis. Besides* B. vulpes*, another species of this genus was molecularly reported in red foxes from Israel and Iraq and provisionally named “*Babesia* sp. MML-2014”; however, no morphological description of this small *Babesia* species was provided, preventing a proper species naming.

**Methods:**

Infection with piroplasmid species was detected and described by microscopy of stained blood smears in one red fox from Southern Italy. Molecular characterization of the *Babesia* sp. and differentiation from *B. vulpes* was performed through PCR amplification of nuclear (*18S rRNA*, ITS1-5.8S-ITS2) and mitochondrial (cytochrome *c* oxidase subunit 1, *cox1*) gene markers, followed by DNA sequencing and phylogenetic analysis. In addition, *Ixodes kaiseri* ticks collected from the infected fox were screened for piroplasmids by PCR.

**Results:**

Sequence comparison of piroplasmids showed 98–99% identity with the undescribed *Babesia* sp. MML-2014 and phylogenetic analyses confirmed that this taxon belongs to the Western group (clade III) and is differentiated by *B. vulpes*. Morphological and morphometric analyses further demonstrated that *Babesia* sp. nov. is a distinct small piroplasm and is characterized by unique Maltese cross forms. Based on the above, we named *Babesia banethi* sp. nov. as a new taxon. In addition, *Babesia* sp. nov. DNA was detected in the intestine of one engorged *I. kaiseri* specimen.

**Conclusions:**

This study provides genetic and morphological findings of *B. banethi* sp. nov. A morphological description with measurements of the parasite forms in red fox erythrocytes, differential diagnosis supplemented by genetic characterization, and the deposition of the holotype in suitable collections have been made in compliance with the ICZN guidelines.

**Graphical Abstract:**

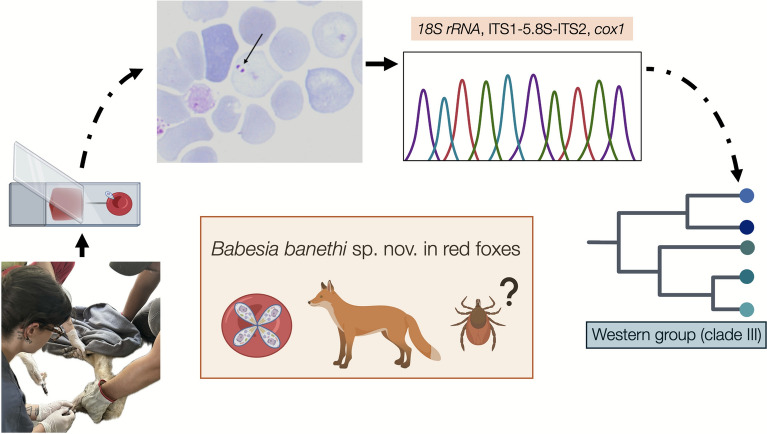

**Supplementary Information:**

The online version contains supplementary material available at 10.1186/s13071-025-07179-y.

## Background

Piroplasmids within the genus *Babesia* are among the most common tick-borne intraerythrocytic protozoa of veterinary and medical concern, with a worldwide distribution and diverse host range [1]. Although more than 100 species have been described in domestic mammals, wildlife, birds, and humans, the remarkable diversity of this heterogeneous genus requires constant updating, with novel species and genotypes assessed through molecular techniques [1]. Within the ten clades described for piroplasmids, based on *18S rRNA* sequences, *Babesia* is phylogenetically characterized as a polyphyletic genus, in four clades, including *Babesia* sensu stricto (i.e., clade X), known as ‘true’ *Babesia*, and other three clades, collectively referred to the *Babesia* sensu lato complex: *Babesia microti*-like group (clade I), Percei group (i.e., clade IV), and Western group (i.e., clade III) [2, 3].

The above molecular and taxonomic distinction between *Babesia* s.s. and *Babesia* s.l. species, reflects crucial biological and morphological features, as the *Babesia* s.s. species display tick-transovarial transmission and have a wide range of host spectrum, with most of them being large piroplasms (i.e., 2.5–5 μm diameter). However, the *Babesia* s.l. complex includes mostly small *Babesia* species (i.e., 1–2.5 μm diameter), without transovarial transmission and higher vertebrate host specificity [2–4].

Compared with *Babesia* s.s. parasites (e.g., *Babesia canis*, *Babesia bigemina*), for which extensive literature is available, data on the biology and pathogenicity of many *Babesia* s.l. species are still very scant. In this context, *Babesia vulpes*, belonging to the *B. microti*-like group (clade I), serves as a key example. Specifically, this parasite was previously classified within the *Theileria* group, as “*Theileria annae*” owing to an equivocal phylogenetic placement [5]. Afterwards, in light of the above incorrect taxonomic position, this parasite has been renamed in several ways, such as “*Babesia* Spanish dog isolate” [6], “*Babesia*-*microti*-like” [7], “*Babesia annae”* [8], *Babesia* (*Theileria*) *annae* [9], and *Babesia* cf. *microti* [10]. In 2019, Baneth and colleagues proposed and established *B*. *vulpes* as the formal species name for this taxon [11]. This parasite primarily circulates among wild canids, especially red foxes of Europe, with molecular prevalence up to 70% in foxes from Spain and Portugal [12, 13]. Dogs, considered accidental hosts, may also be infected, displaying severe disease with pale mucous membranes, anemia, anorexia, and lethargy [14], which suggests that this parasite may be diagnosed in domestic animals living in sympatry with foxes [8, 15].

Besides *B. vulpes*, another species of this genus was molecularly reported in both red foxes and golden jackals from Israel [16] and Iraq [17] and was provisionally named “*Babesia* sp. MML-2014” by the first author’s initials of the above study [16]. Based on the sequencing of *18S* (1490 bp) and *ITS2* (445 bp) genes, *Babesia* sp. MML-2014 showed the highest molecular identity with *Babesia lengau* sequences (clade III, Western group), within the *Babesia* s.l. complex [4, 16]. However, this small *Babesia* species has never been detected by light microscopy in either red fox or golden jackal blood, therefore preventing morphological description and proper species naming.

In this study, we provide morphological features and morphometric measurements of intraerythrocytic “*Babesia* sp. MML-2014” parasites, as well as a thorough molecular analysis of multiple genetic markers (i.e., *18S rRNA*, ITS1-5.8S-ITS2, and *cox1*) to differentiate it from *B. vulpes*. Based on the above genetic and morphological evidence, we herein name the “*Babesia* sp. MML-2014” species derived from a sick red fox (*Vulpes vulpes*) from southern Italy.

## Methods

### Animal sampling and blood smear evaluation

In January 2025, an adult female fox (i.e., #Fox1) was brought to the wildlife rescue center in Bitetto (Apulia, Italy) following a car accident; health status and laboratory test parameters, including complete blood cell count (CBC) and serological biochemical parameters, were included in the individual clinical file. Blood sampling was performed without sedation, using only physical restraint.

**Fig. 1:**
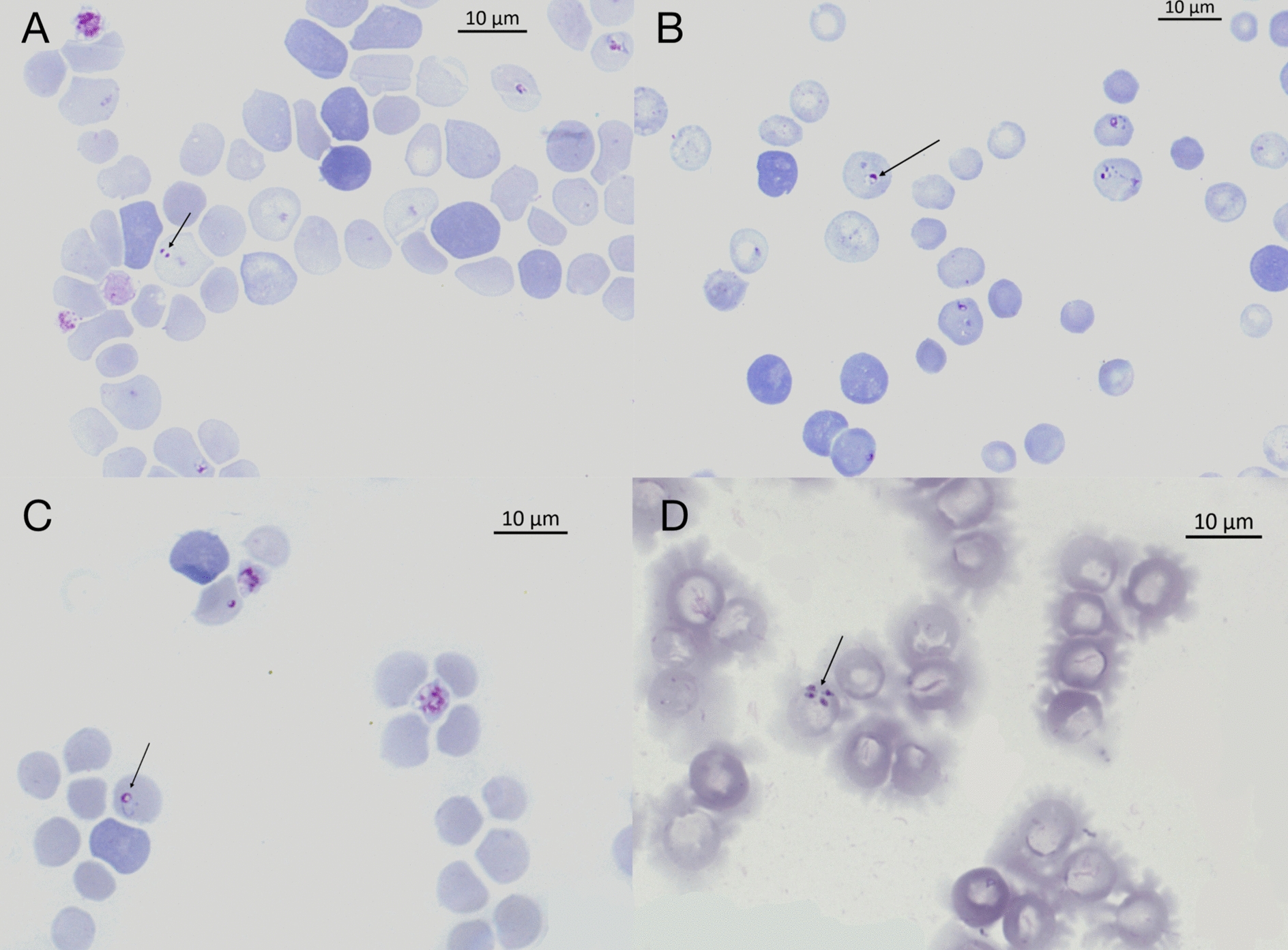
*Babesia banethi* sp. nov. type-material in blood smears from #Fox1. Diff Quik staining, 100 × magnification. **A** Parasite holotype: merozoites with dense chromatin nuclei located in the rounded poles of their elongated forms; **B** Parasite paratype: merozoites with eccentric nucleus located at one pole; **C** Parasite paratype: merozoites with elongated nucleus adhering to the inner part of the body; **D** Parasite paratype: four merozoites presented as tetrad shapes or Maltese cross

Blood smears, stained with Romanowsky solutions, were visualized by oil immersion microscopy at 1000 × magnification (Axioscope 5, Carl Zeiss Microscopy GmbH, Germany); piroplasm parasites were detected in the erythrocytes, and images were acquired with a digital camera (Axiocam 305 color, Carl Zeiss) (Fig. 1). Images were processed using ZEN 3.1 Pro software (Carl Zeiss), which was also used for morphometric measurements (e.g., length and width) of intraerythrocytic *Babesia* spp. forms.

### Molecular detection of *Babesia* spp.

**Fig. 2:**
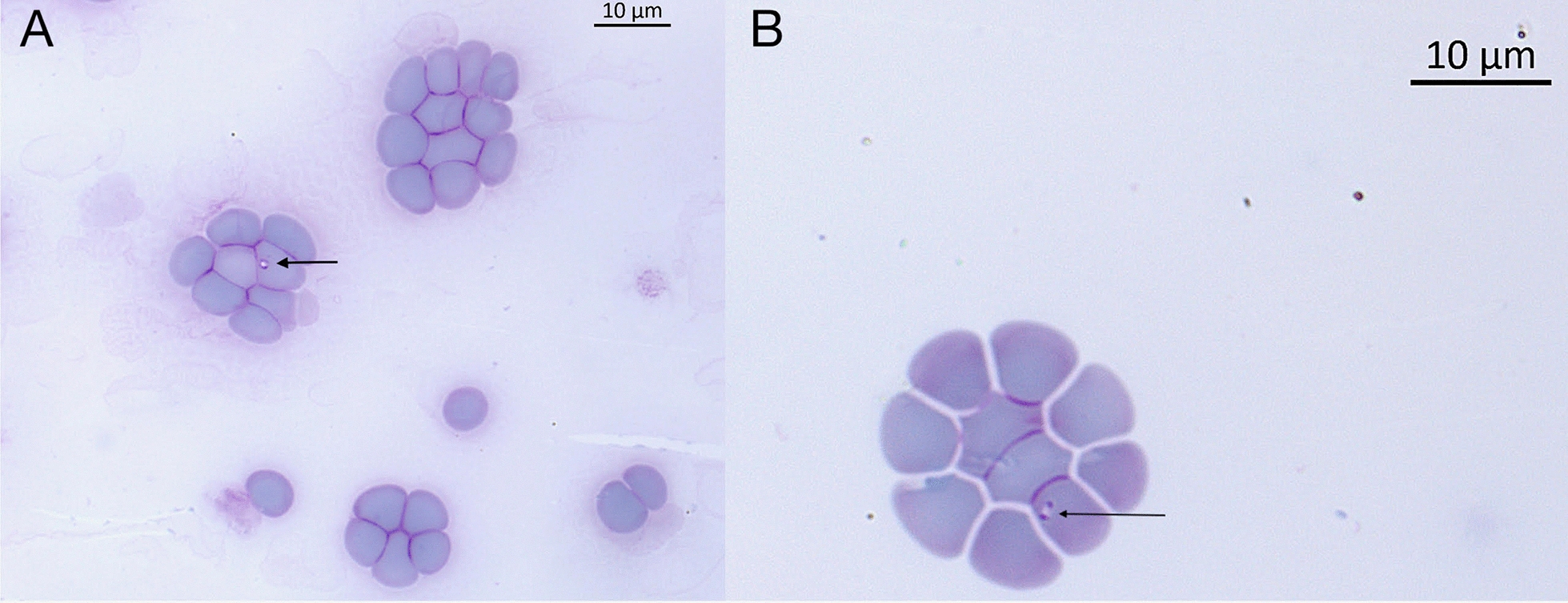
*Babesia vulpes* merozoites retrieved from #Fox2. Diff Quik staining, 100 × magnification. **A** Oval-shaped merozoite resembling a ring form. **B** Elongated merozoite with nuclei located at the two poles

DNA was extracted from 200 μl of EDTA blood using a commercial kit (QIAamp DNA Blood Tissue, Qiagen, Hilden, Germany) following the manufacturer’s protocol. *Babesia* sp. molecular detection was performed using four conventional PCRs (cPCR) targeting *18S rRNA*, ITS1-5.8S-ITS2, cytochrome *c* oxidase subunit 1 (*cox1*), and cytochrome *b* (*cytB*) partial sequence, yielding different-sized amplicons (Table 1). Primers employed and amplification conditions are reported in Table 1. The same PCR protocols were applied to amplify blood DNA from a healthy fox previously assessed as infected by *B. vulpes* (i.e., #Fox2) by both blood smear evaluation (Fig. 2) and cPCR targeting *18S rRNA* gene (RLBF/R, ~510 bp).

**Table 1:** Amplification protocols (i.e., target genes, primers and conditions) employed for *Babesia* spp. detection and characterization in fox blood

Target locus/gene	Primers	Primer sequence	Fragment length (bp)	Annealing temperature and time	Number of cycles	Reference
*18S rRNA*	BT1-F	GGTTGATCCTGCCAGTAGT	1036	68 °C for 1 min	40	[18]
	BT2-R	CTTCTGCAGGTTCACCTACG				
*18S rRNA*	RLB_F	GAGGTAGTGACAAGAAATAACAATA	520	54 °C for 30 s	39	Adapted from [40]
	RLB_R	TCTTCGATCCCCTAACTTTC				
ITS1-5.8S-ITS2	ITS_F	GTGAACCTTATCACTTAAAGG	940	52 °C for 60 s	40	Adapted from [41]
	ITS_R	TTCRCTCGCCGYTACT				
Cytochrome *c* oxidase subunit* 1* (*cox1*)	Cox1F133	GGAGAGCTAGGTAGTAGTGGAGATAGG	1023	66 °C for 60 s	45	[11]
	Cox1R1130	GTGGAAGTGAGCTACCACATACGCTG				
Cytochrome *b* (*cytb*)	COB-F	CCATAGCAATTAATCCAGCTA	550	54 °C for 30 s	35	[41]
	COB-R	ACCTTGGTCATGGTATTCTGG

Amplified PCR products were visualized by gel-electrophoresis in 2% agarose gel containing GelRed nucleic acid gel stain (VWR International PBI, Milan, Italy) and viewed on a ChemiDoc documentation system (BIORAD, Hercules, California, USA). All the positive cPCR products were purified and sequenced in both directions using the same primers, employing the Big Dye Terminator v.3.1 chemistry in a 3130 Genetic analyzer (Applied Biosystems, Foster City, California, USA) in an automated sequencer (ABI-PRISM 377). Nucleotide sequences were edited, aligned, and analyzed using the Geneious platform version 9.0 (Biomatters Ltd., Auckland, New Zealand) and compared with available sequences in the GenBank database using the Basic Local Alignment Search Tool (BLAST; http://blast.ncbi.nlm.nih.gov/Blast.cgi) for species identification.

### Amplification of a larger fragment of *Babesia* spp. *18S rRNA* gene

For the sequencing of the *18S rRNA* gene long fragment, DNA was extracted from blood samples of #Fox1 and #Fox2, using MagMAX CORE Nucleic Acid Purification Kit (ThermoFisher Scientific, Waltham, CA, USA) by TANBead Maelstrom 4810 (Taiwan Advanced Nanotech, Taoyuan, Taiwan) automated nucleic acid extraction system at the Istituto Zooprofilattico Sperimentale della Puglia e della Basilicata. Both DNA samples (i.e., #Fox1 and #Fox2) were tested by cPCR targeting a long fragment of the *18S rRNA* gene (1036 bp) [18] (Table 1). All amplicons were visualized in the QIAxcel Advanced System capillary gel electrophoresis system (QIAGEN, Germany) using the QIAxcel DNA High Sensitivity Kit. The amplicons were then purified using ExoSAP-IT according to the supplier’s recommendations (GE Healthcare, Chicago, IL, USA). Sequence reactions were performed using BigDye 3.1 Ready reaction mix (Life Technologies, Thermo Fischer Scientific, Carlsbad, CA, USA) according to the manufacturer’s instructions and sequenced using ABI 3130 automated sequencer (Applied Biosystems, Waltham, MA, USA). Sample-specific sequence chromatogram files were analyzed using Bionumerics 7.6 software (Applied Maths, Sint-Martens-Latem, Belgium) for editing and assembling. The obtained *18S rRNA* partial sequences were used as query sequences on the NCBI BLASTn tool (https://blast.ncbi.nlm.nih.gov/Blast.cgi, accessed in June 2025, “nr” database), to obtain taxonomic assignments. The partial *18S rRNA* sequences of both *Babesia* sp. and *B. vulpes* were aligned and compared with each other.

### Long-read metagenomic sequencing and analysis of *cox1* gene

The extracted genomic DNA of blood from the #Fox1 sample was additionally subjected to long-read metagenomic sequencing. DNA quantification was evaluated in a Qubit 3.0 Fluorometer using a Qubit dsDNA High Sensitivity (HS) kit (Invitrogen, Life Technologies, Milan, Italy), according to the manufacturer’s instructions. The library was prepared using the Ligation Sequencing Kit (SQK-LSK109), purified using Agencourt AMPure XP magnetic beads (Beckman CoulterTM, Indianapolis, IN, USA), and sequenced on a MinION MK1C device (Oxford Nanopore Technologies, ONTTM, Oxford, UK) with R.10.4.1 flowcell, Guppy v6.5, and “fast” as base calling mode. The raw Fastq files were initially merged and quality checked by NanoPlot v.1.42.1 and filtered by read quality and length (–min_length '500' –min_mean_q '5.0') by filtlong v0.2.1. Filtered reads were classified by using the Kraken2 tool v2.1.3 with default parameters and ‘2025–01-04T202436Z_standard_prebuilt_core_nt’ as the reference database. Thus, host genome and contaminant DNA were depleted by using Krakentools’ “extract kraken reads by ID” and only reads with confident taxonomic assignment to Babesiidae (NCBI taxonomy ID: 32,594) were retained. Necat long-read assembly tool v0.0.1 was used to polish and assemble selected reads, while Medaka v1.7.2 with error model ‘r1041_e82_400bps_fast_g615’ was used for assembly sequence correction. The refined sequence was searched for similarity by means of BLASTn (‘core_nt’ database); alignment of the query input sequence and 100 matching subject sequences was downloaded and reviewed through BioEdit version 7.2.5. A region homologous to the *cox1* gene of *Babesia duncani* mtDNA (NCBI Id: NC_039721.1) was carefully inspected: CDS start and stop codons were identified, obtaining full-length CDS and amino acid *cox1*sequences, with compatible length to *B. duncani* orthologs. The discovered amino acid sequence was used as a query for BLASTp similarity search on the NCBI nr database; the Top 100 high-score matching sequences were realigned together with the query *cox1* by Clustal Omega (https://www.ebi.ac.uk/jdispatcher/msa/clustalo, accessed on 03/12/2025).

### Phylogenetic analysis

Phylogenetic analyses were performed to determine the evolutionary placement of the novel identified species of the genus *Babesia*. Three genetic markers were analyzed: the *18S rRNA* gene (1036 bp), the *ITS1-5.8S-ITS2* (940 bp), and the *cox1* (875 bp). Sequences obtained in this study were aligned with homologous sequences of other *Babesia* species retrieved from GenBank. Multiple sequence alignments were conducted using MUSCLE, and maximum likelihood (ML) trees were constructed in MEGA12 [19]. The best evolutionary model for each dataset was assessed, and the robustness of the tree topology was evaluated through 8000 bootstrap replicates.

### Tick collection

At general examination, ticks were collected from #Fox1 and morphologically identified on the basis of dichotomous keys, as well as original descriptions [20–22]. Engorged female ticks were dissected, and cytological slides were prepared with tick organs (i.e., intestine, hemolymph, salivary glands, and ovary) and stained with Giemsa solution at 1:20 dilution; slides were observed under light microscopy for the detection of *Babesia* spp. life stages, whereas the remaining part of the tick organs was stored for further analyses. To molecularly confirm tick morphological identification, DNA was extracted from all ticks collected (both whole ticks and tick organs retrieved from engorged ones), using a commercial kit (DNeasy Blood & Tissue Kit, Qiagen, Hilden, Germany) and was further tested by two cPCRs targeting *cox1* and *16S* genes [23, 24] (Table 1). Piroplasmids were also searched for by *18S rRNA* amplification (RLBF/R, ~510 bp, see above) (Table 1), and sequences from positive DNA amplicons were identified using BLAST, as described above.

## Results

Family Babesiidae Poche, 1913.

Genus *Babesia* Starcovici, 1893.

*Babesia banethi* sp. nov. Otranto, Carbonara, Šlapeta.

*Type-host* Red fox (*Vulpes vulpes* Linnaeus, 1758).

*Other hosts* Golden jackal (*Canis aureus* Linnaeus, 1758).

*Type-locality* Bitetto, Southern Italy (41°02′N, 16°45′E).

*Other localities* Israel [16], Iraq [17].

*Type-material* A stained thin blood smear from an adult female fox (i.e., #Fox1) containing the holotype (Fig. 1a, identified by an arrow) was deposited at the IPCAS Institute of Parasitology, Academy of Sciences of the Czech Republic, České Budějovice, Czech Republic (Reference collection number: IPCAS Pro 89).

Blood smears containing paratypes were deposited at the Parasite Collection IPCAS Institute of Parasitology, Academy of Sciences of the Czech Republic, České Budějovice, Czech Republic (Reference collection number: IPCAS Pro 89) (Fig. 1b,c,d).

### Vector: unknown

Representative DNA sequences: present study (GenBank: PX387919, *18S rRNA* of *B. vulpes* from #Fox2; PX387920, *18S rRNA* of *B. banethi* sp. nov. from #Fox1; PX400667, ITS1-5.8S-ITS2 of *B. vulpes* from #Fox2; PX400666, ITS1-5.8S-ITS2 of *B. banethi* sp. nov. from #Fox1; PX394400, *cox1* of *B. vulpes* from #Fox2; PX394401, *cox1* of *B. banethi* sp. nov. from #Fox1);

[16, 17] (GenBank: MG461685-6; MK957184; *18S rRNA*).

ZooBank registration: to comply with the regulations set out in article 8.5 of the amended 2012 version of the International Code of Zoological Nomenclature (ICZN) [25], details of the new species have been submitted to ZooBank. The Life Science Identifier (LSID) of the article is urn:lsid:zoobank.org:pub:01268571-BEA2-4218-80E3-A8CCDCF7A918. The LSID for the new name *Babesia banethi* is urn:lsid:zoobank.org:act:F914E216-039A-417A-9CC1-CB60FF72381A.

Etymology: named in honor of Professor Gad Baneth for his significant contributions to the study of protozoan parasites, his mentorship in parasitological research, and his dedication to international scientific collaboration. The species epithet *banethi* is a noun in apposition.

### Description

Merozoites [Measurements based on 50 parasites; see Fig. 1.]. Round to oval-shaped merozoites with an eccentric basophilic and pleomorphic nucleus adhering to the parasite’s inner limits. Merozoites measure on average 1.9 ± 0.5 µm in length and 1.2 ± 0.5 µm in width and are present as single or four parasites in the erythrocyte (i.e., tetrad or Maltese cross).

### Molecular identification

Although both long and short fragments of the *18S rRNA* gene were obtained, only the long fragment (1036 bp) was used for nucleotide comparison and phylogenetic reconstruction, as the regions overlapped longer sequences, providing more informative sites. Attempts to amplify the c*ytB* fragment by cPCR were unsuccessful, as no specific product could be obtained from either #Fox1 or #Fox2 blood DNA samples; in addition, *cox1* amplification through cPCR (1023 bp, Cox1F133/Cox1R1130) was possible only for *B. vulpes* from #Fox2, meanwhile, the *cox1* sequence of* B*. *banethi* sp. nov. from #Fox1 was achieved by the above metagenomic analyses.

**Fig. 3:**
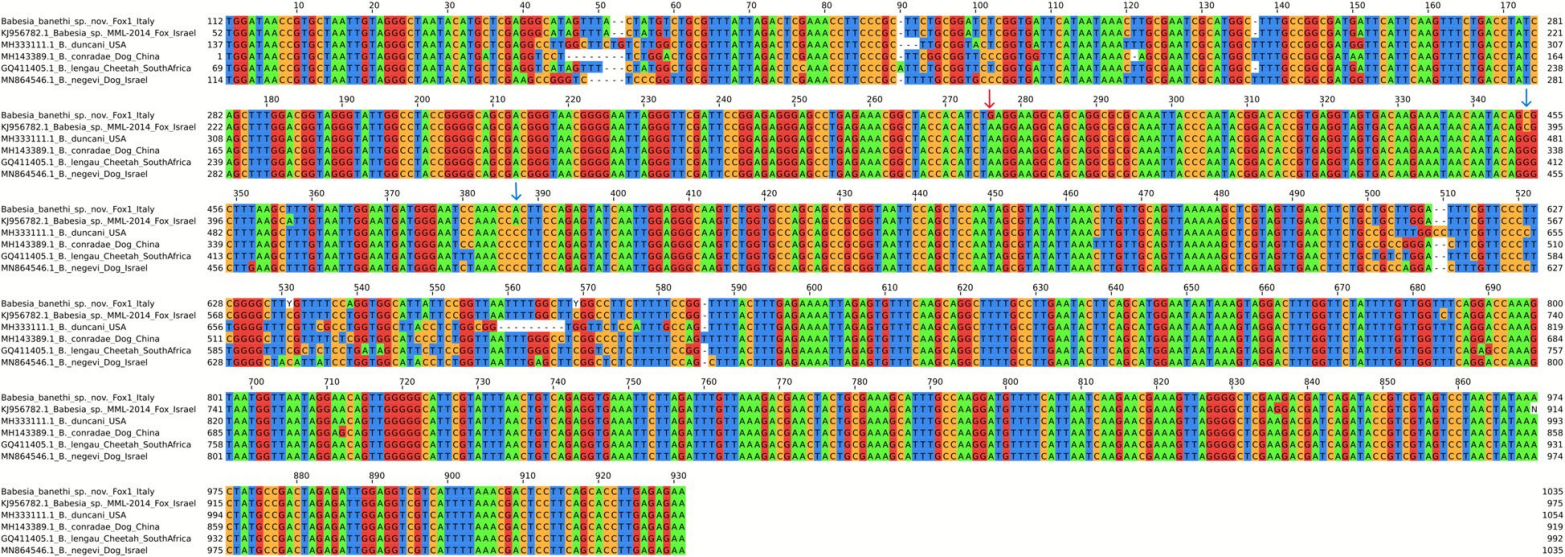
Multiple sequence alignment of the *18S rRNA* nucleotide sequences of *Babesia banethi* sp. nov. from #Fox1 (Italy), *Babesia* sp. MML-2014 (GenBank accession no. KJ956782.1) and reference sequences belonging to the same clade (i.e., Western group, clade III). The red arrow indicates a position conserved within the Western group but distinct in *B. banethi* sp. nov. The blue arrows indicate positions conserved within the Western group but differing in both *B. banethi* sp. nov. and *Babesia* sp. MML-2014. The alignment was generated and visualized using Jalview v2.11.5.0

Pairwise comparison of the *18S rRNA* gene nucleotide sequences (1036 bp) obtained from fox blood samples revealed 99.49% identity between the sequence of *B*. *banethi* sp. nov. from #Fox1 and *Babesia* sp. MML-2014 (GenBank KJ956782.1), with single nucleotide polymorphisms (Fig. 3; Table 2) at positions 383 (A > G), 464 (A > T), 636 (C > Y), 644 (T > A), 674 (C > Y), 788 (C > T), 940 (G > A), and 974 (N > A). *Babesia banethi* sp. nov. and *Babesia* sp. MML-2014 showed nucleotide variations in the *18S rRNA* gene when compared with the Western group *Babesia* spp., specifically at positions 347 (G > C) and 387 (C > A) of the multiple sequence alignment (MSA) (Fig. 3). In addition, *B. banethi* sp. nov. exhibited a single nucleotide polymorphism (SNP) at position 276 (A > G) of the MSA when compared with all the other *Babesia* spp., including *Babesia* sp. MML-2014. When comparing the *18S rRNA* sequences of *B*. *banethi* sp. nov. (#Fox1) and *B. vulpes* (#Fox2), they differed by 11.15% nucleotide identity.

**Table 2:** Intra- and inter-specific variation percentage between *18S rRNA* nucleotide sequences of *Babesia banethi* sp. nov. and other *Babesia* spp. from clade III

No	Species	ID	1	2	3	4	5	6
1	*Babesia banethi* sp. nov	#Fox1						
2	*Babesia* sp. MML-2014	KJ956782.1	0.543					
4	*Babesia lengau*	GQ411405.1	2.606	2.711				
3	*Babesia conradae*	MH143389.1	3.607	3.821	4.035			
5	*Babesia duncani*	MH333111.1	4.391	4.605	4.386	4.410		
6	*Babesia negevi*	MN864546.1	4.897	5.000	4.669	3.813	3.842	

Analysis of the ITS1-5.8S-ITS2 nucleotide sequences (940 bp) generated from the same fox blood samples showed a 98.44% identity between the sequence of *B*. *banethi* sp. nov. from #Fox1 and *Babesia* sp. MML-2014 (GenBank KR709304.1). Nucleotide substitutions were identified at positions 229 (A > G), 235 (G > T), 243 (N > G), 259 (T > C), 267 (C > T), 288 (N > T), and 295 (A > T), along with a thymine deletion at position 254 of the alignment (Sup. Figure 1; Table 3). Overall, the ITS1-5.8S-ITS2 sequences from #Fox1 (*B*. *banethi* sp. nov.) and #Fox2 (*B. vulpes*) differed by 45.15% of nucleotide identity between them.

**Table 3:** Intra- and inter-specific variation percentage between ITS1-5.8S-ITS2 nucleotide sequences of *Babesia banethi* sp. nov. and other *Babesia* spp. from clade III

No	Species	ID	1	2	3	4
1	*Babesia banethi* sp. nov	#Fox1				
2	*Babesia* sp. MML-2014	KR709304.1	1.56			
3	*Babesia duncani*	AY965741.1	16.24	10.30		
4	*Babesia conradae*	AY965738.1	17.43	14.51	19.66	

Pairwise comparison of the *cox1* nucleotide and amino acid sequences (Supplementary Fig. 2, 3; Table 4) between *B. banethi* sp. nov. (#Fox1) and *B. duncani* (GenBank: NC_039721.1 and YP_009529218.1, respectively) revealed sequence identities of 79.9% (nucleotide) and 78.98% (amino acid). When compared with the other sequences of *Babesia* spp. from the Western group, in which these residues are conserved, *B. banethi* sp. nov. showed nine amino acid substitutions in the MSA, Ser132Ala, Ser151Ala, Ala181S, Ile212Val, Val324Ile Leu370Val, Leu377Ile, Ile412Val, and Val415Ile in the MSA. Comparison of *B. banethi* sp. nov. (#Fox1) and *B. vulpes* (#Fox2) sequences showed lower identity values for both nucleotide (67.57%) and amino acid (64.06%) sequences.

**Table 4:** Intra- and inter-specific variation percentage between *cox1* nucleotide sequences of *Babesia banethi* sp. nov. and other *Babesia* spp. from clade III

No	Species	ID	1	2	3	4
1	*Babesia banethi* sp. nov	#Fox1				
2	*Babesia negevi*	MN876837.1	20.1			
3	*Babesia duncani*	NC_039721.1	23.1	23.8		
4	*Babesia conradae*	KC207826.1	26.1	24.5	29.6	

Intra- and inter-specific variation rates between *B*. *banethi* sp. nov. and reference sequences belonging to the same clade (i.e., Western group, clade III) [2] are detailed in Tables 2, 3, and 4.

New DNA sequences from the *Babesia* spp.-infected foxes were deposited as new accession numbers in the GenBank database, as follow: PX387919 (*18S rRNA* of *B. vulpes* from #Fox2), PX387920 (*18S rRNA* of *B. banethi* sp. nov. from #Fox1), PX400667 (ITS1-5.8S-ITS2 of *B. vulpes* from #Fox2), PX400666 (ITS1-5.8S-ITS2 of *B. banethi* sp. nov. from #Fox1), PX394400 (*cox1* of *B. vulpes* from #Fox2), and PX394401 (*cox1* of *B. banethi* sp. nov. from #Fox1).

### Molecular phylogeny

Phylogenetic analyses based on the ITS1-5.8S-ITS2, *18S rRNA* and *cox1* sequences, consistently supported the presence of two genetically distinct *Babesia* species in foxes from southern Italy. The *Babesia* sequence retrieved from #Fox1 formed a separate and well-supported lineage across all the analyses, distinct from *B. vulpes* and other described *Babesia* spp., suggesting the presence of a novel species within the Western group (clade III) [2]. Differently, the *Babesia* sequence retrieved from #Fox2 was consistently placed within the *B. vulpes* clade in all phylogenetic trees and had previously been identified as *B. vulpes* by cPCR, confirming its classification.

**Fig. 4:**
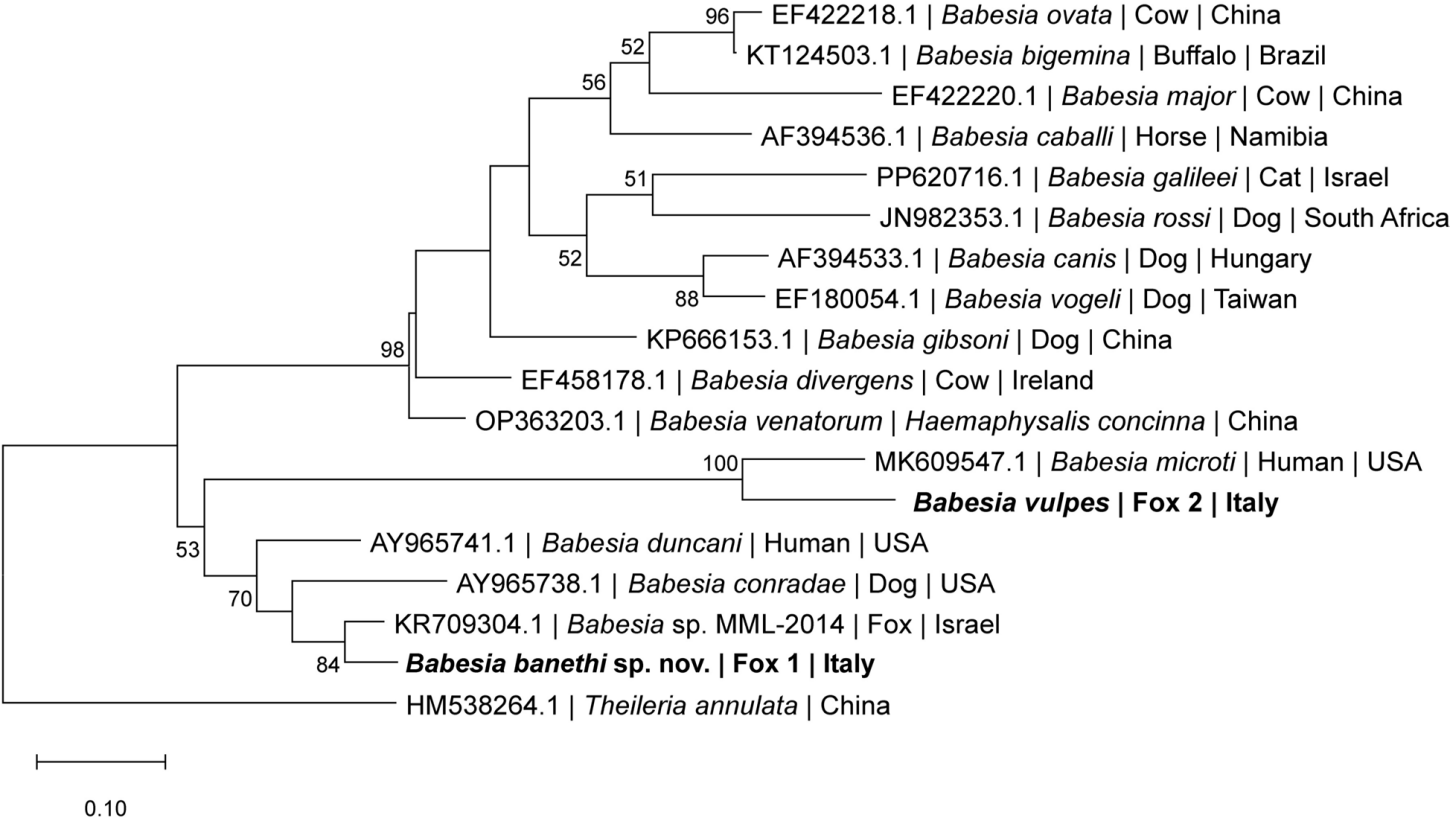
Phylogenetic tree of ITS1-5.8S-ITS2 sequences of *Babesia* species (509 bp). Sequences of *Babesia banethi* sp. nov. and *Babesia vulpes* from foxes in this study (bold) are compared with partial ITS1-5.8S-ITS2 sequences of other relevant *Babesia* species available in GenBank. GenBank accession numbers, host species, and country of origin are indicated for each sequence. The tree was inferred using the maximum likelihood method under the Kimura 2-parameter + G + I model. This analysis included 18 nucleotide sequences. *Theileria annulata* was used as the outgroup. The scale bar indicates the number of nucleotide substitutions per site. Bootstrap values lower than 50 were not shown in the figure

The ITS1-5.8S-ITS2 phylogeny (Fig. 4) showed that *Babesia* sp. from #Fox1 grouped in a distinct clade, separately from *B. vulpes*, *B. microti*, and other mammalian *Babesia* spp., while *Babesia* sequence from #Fox2 grouped tightly with *B. microti.* Specifically, the ITS2 amplified from #Fox2 DNA exhibited a 99.40% identity with *B. vulpes* (GenBank PQ415088.1).

**Fig. 5:**
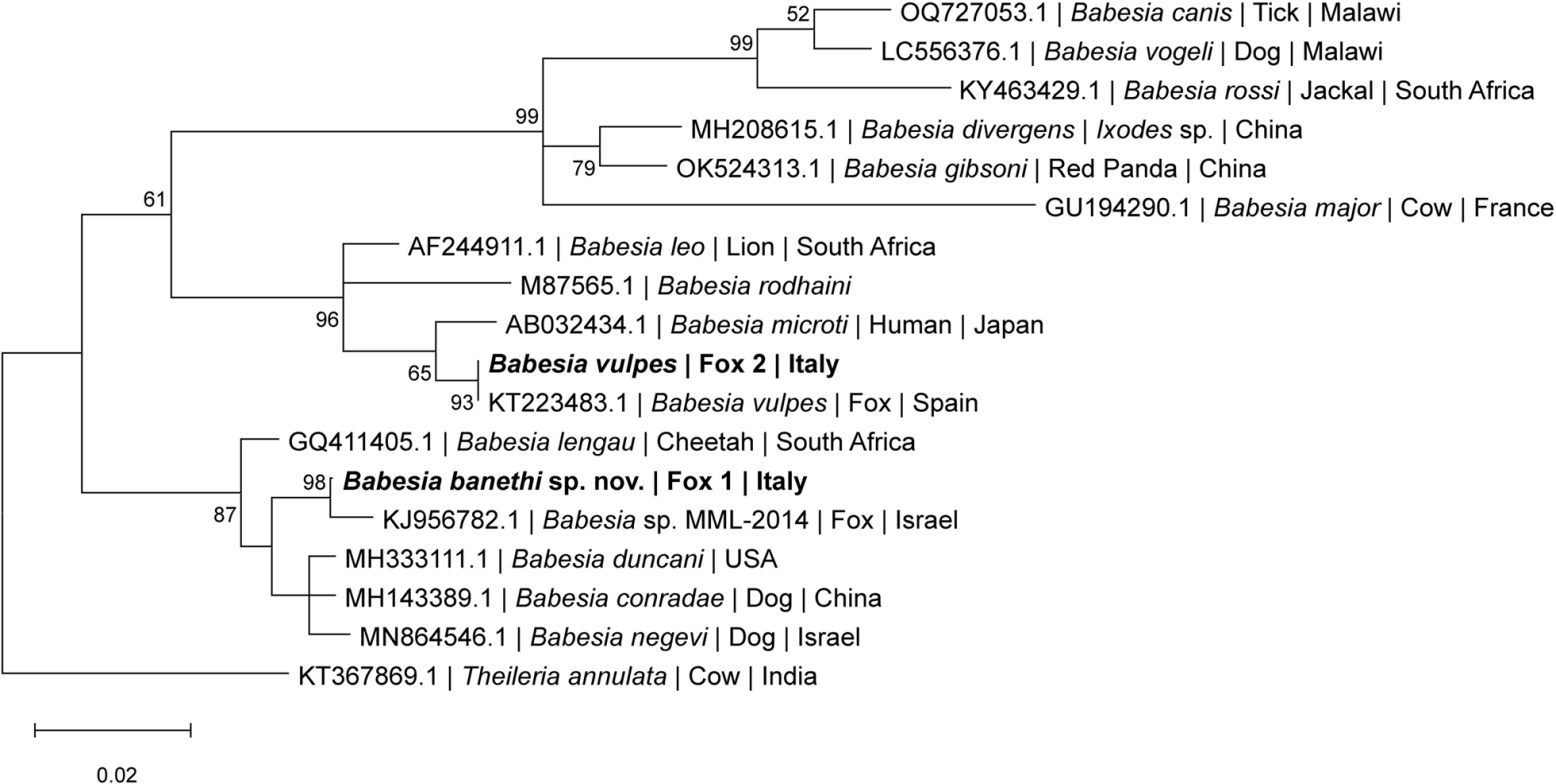
Phylogenetic tree of *18S rRNA* (611 bp) sequences of *Babesia* species. Sequences of *Babesia banethi* sp. nov. and *Babesia vulpes* from foxes in this study (bold) are compared with *18S rRNA* sequences of other relevant *Babesia* species available in GenBank. GenBank accession numbers, host species, and country of origin are indicated for each sequence. The tree was inferred using the maximum likelihood method under the Tamura-Nei + G + I model. *Theileria annulata* was used as the outgroup. The scale bar indicates the number of nucleotide substitutions per site. Bootstrap values lower than 50 were not shown in the figure

The *18S rRNA* phylogeny (Fig. 5) confirmed the placement of *B. vulpes* sequence herein obtain (#Fox2) within the *B. vulpes* clade, this sequence being 99.34% identical with *B. vulpes 18S rRNA* partial sequence (GenBank KJ871351.1). However, the sequence of *B. banethi* sp. nov. (#Fox1) clustered separately, as a sister group to *Babesia* sp. MML-2014, confirming its phylogenetic distinctiveness across the long sequences of *18S rRNA* gene.

**Fig. 6:**
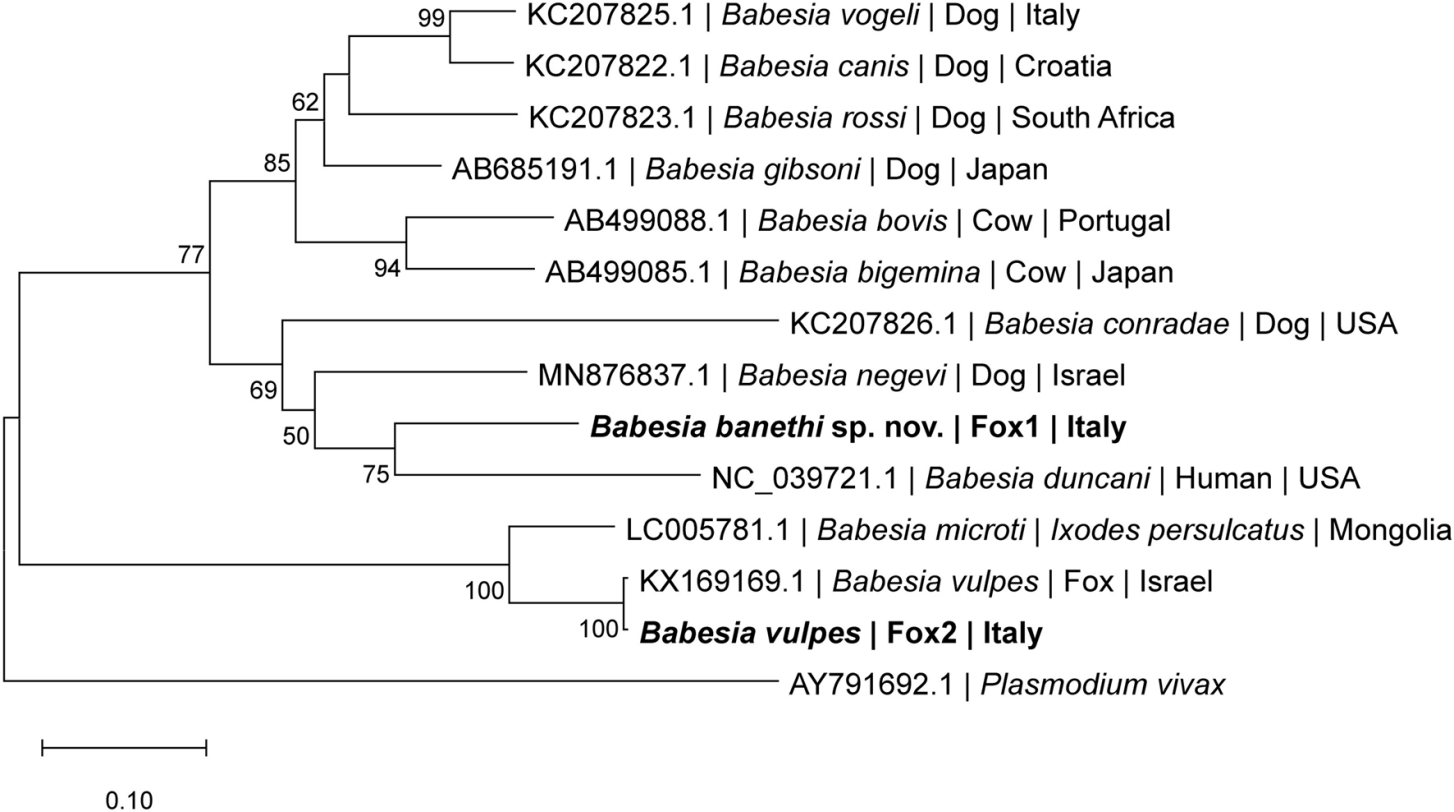
Phylogenetic tree of *cox1* nucleotide sequence of *Babesia* species (875 bp). Maximum likelihood phylogeny based on *cox1* gene sequences, including *Babesia vulpes* and *Babesia banethi* sp. nov. from this study (bold) and other related *Babesia* spp. sequences from GenBank. GenBank accession numbers, host species, and country of origin are indicated for each sequence. The tree was inferred using the maximum likelihood method under the GTR + G + I model. *Plasmodium vivax* was used as the outgroup. The scale bar indicates the number of nucleotide substitutions per site. Bootstrap values lower than 50 were not shown in the figure

Phylogenetic analysis of the *cox1* nucleotide sequences showed that *B. banethi* sp. nov. (#Fox1) clustered most closely with *B. duncani* (GenBank: NC_039721.1), and this clade subsequently clustered with *Babesia conradae *(GenBank: KC207826.1), both reported in the USA (Fig. 6). As observed for the other genetic markers, the *cox1* sequence retrieved from #Fox2 DNA also showed phylogenetic proximity to *B. vulpes*, which was reported in Israel (GenBank: KX169169.1). Accordingly, sequences of *B. vulpes* obtained from #Fox2 had high nucleotide (99.54%) and amino acid (99.29%) identity with the sequence of *B. vulpes* from Israel (GenBank: KX169169.1).

Overall, the molecular confirmation of *B. vulpes* infection in #Fox2 and the consistent molecular divergence of *B. banethi* sp. nov. from #Fox1 across multiple markers support the classification of the latter as a distinct taxon, representing a novel *Babesia* species within the Western clade.

### Morphological differential diagnosis

Intraerythrocytic parasites presented as round to oval shaped, and an eccentric, basophilic-staining pleomorphic nucleus, which was conspicuous in some parasites. Specifically, some merozoites presented with basophilic staining dense chromatin nuclei located in the rounded poles of their elongated forms (Fig. 1a); others presented an eccentric nucleus located at one pole of the oval shaped merozoite (Fig. 1b); some parasites had an elongated nucleus adhering, partially or totally, to the inner part of the circular shaped merozoite (Fig. 1c). Of the 50 parasites measured, *n* = 38 were presented as single parasite and occupied only a small portion of the erythrocyte, resembling the ring shapes described for *B. vulpes* [11] (Fig. 1a,b,c); *n* = 12 parasites were presented as tetrad shapes (Fig. 1d), resembling the Maltese cross described for *B. duncani* and *B. conradae* [26–28]. The merozoites of *B. banethi* sp. nov., measuring on average of 1.9 × 1.2 ± 0.5 µm, are distinctly bigger than the merozoites of *B. vulpes* (i.e., 1.33 × 0.98 μm, [11]; 1.6 × 1.1 ± 0.3, measurements based on five parasites retrieved from #Fox2; Fig. 2); as well as, differently from *B. vulpes, B. banethi* sp. nov. presented Maltese cross. In addition, the merozoites of *B. banethi* sp. nov. are smaller in size than the large canine *Babesia* spp., measuring 4.5–5.0 × 2.0–2.5 μm [29], but similar to the small canine *B. gibsoni* (1.9 × 1.2 μm) [26] and smaller than the ring forms of the canine *B. conradae* (2.2 × 1.85 μm) [28] and the bat *Babesia vesperugini* (1.5 – 6.5/ μm in length) [30]. However, the merozoites of *B. banethi* sp. nov. are shorter than the ring forms of the human *B. duncani* (i.e., 2.4 ± 0.18 μm in diameter) [27] but identical to those of *B. lengau* (1.91 × 1.1 μm) [31], infecting wild felids from South Africa. The above comparisons indicate that *B*. *banethi* sp. nov. is a distinct form consistent with the small-form piroplasms of canids, wild felids and humans.

### Clinical findings in infected fox

On physical examination, #Fox1 presented with mild pallor of the oral mucous membranes. Body condition score was normal, as well as the remainder of the physical examination. Abnormalities on serum biochemistry included increased of creatine phosphokinase (CPK; 777; range: 37–623 IU/L), aspartate aminotransferase (AST; 218; range: 9–118 UI/L), and alanine aminotransferase activities (ALT; 611; range: 39–373 UI/L); along with hyperglobulinaemia (4.6; range: 1.5–3.9 g/dL) and reduced albumin/globulin ratio (A/G; 0.5; range: 0.6–1.3). The complete blood cell count identified the presence of rouleaux and microcites; leukocytosis (13.4 × 1000/L leukocytes [range: 2.5–9.9 × 1000/L] with an increased number of segmented neutrophils (11 × 1000/L [range: 0.5–5.9 × 1000/L]) and monocytes (1072; range: 0–500 mg/μL). A blood smear examination showed infection of red blood cells with piroplasmid organisms interpreted as *Babesia* sp., and blood was taken for PCR to verify and genetically characterize the infecting organism, as above described. #Fox1 did not receive any treatment for *Babesia* infection. However, after about 2 weeks of cage rest and supportive therapy, the animal showed a marked improvement in its physical condition and was released back into nature.

### Ticks

**Fig. 7:**
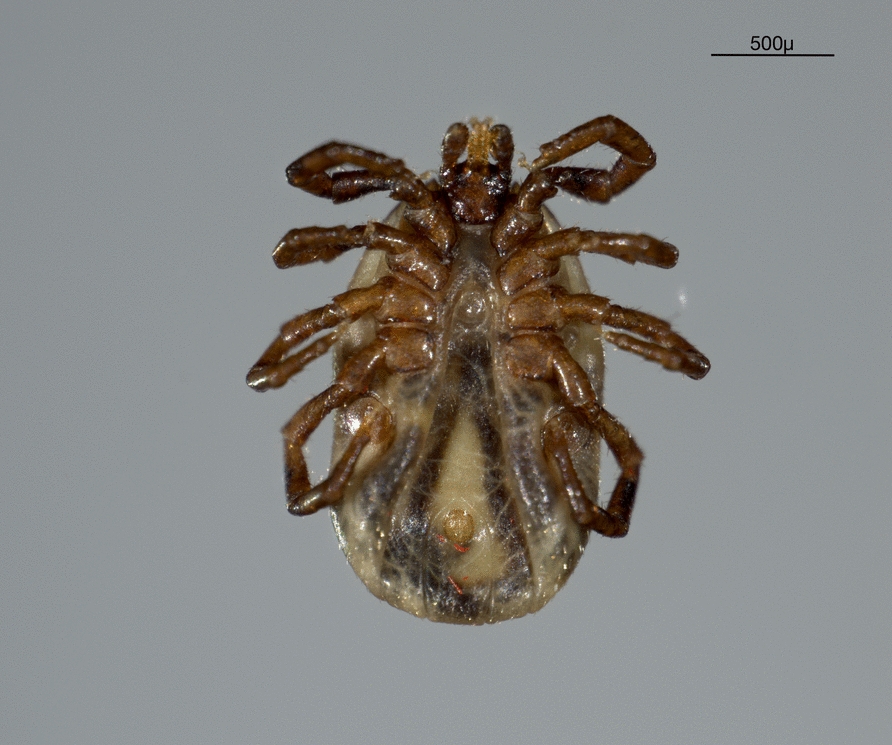
Ventral view of adult female specimen of *Ixodes kaiseri* collected from a fox (#Fox1) infected by *Babesia banethi* sp. nov

Overall, *n* = 14 adult female ticks were collected alive from #Fox1; four tick specimens were engorged. Ticks were morphologically and molecularly identified as *Ixodes kaiseri* (Fig. 7) (i.e., 16S: 99.5% nucleotide identity with GenBank sequence accession MK946451; *cox1*: 100% nucleotide identity with GenBank sequence accession MK946448). No *Babesia* spp. life stages were observed in tick organs, although *B*. *banethi* sp. nov. was amplified (i.e., 99.7% nucleotide identity with GenBank sequence accession n. KJ956782, *Babesia* sp. MML-2014) from the intestine, after the dissection of one engorged *I. kaiseri* after 14 days from the detachment. The remaining DNA samples of tick organs and whole ticks scored negative for *Babesia* spp.

## Discussion

This study presents *B. banethi* sp. nov. as a new taxon by fulfilling the ICZN guideline requirements for a new species [25]. A morphological description with measurements of the parasite forms in red fox erythrocytes, differential diagnosis supplemented by genetic characterization and the deposition of the holotype in suitable collections have been made in compliance with the ICZN guidelines [25].

The placement of *B. banethi* sp. nov. in the genus *Babesia*, and its segregation in the *Babesia* s.l. complex (Western group) is derived from the pairwise comparison and phylogenetic analysis of the *18S rRNA*, ITS1-5.8S-ITS2 and *cox1* sequences. Accordingly, phylograms based on nuclear and mitochondrial markers, revealed the presence of two distinct *Babesia* species infecting foxes from southern Italy: *B. vulpes*, previously reported as the dominant piroplasm in European foxes [12, 13], and a genetically distinct lineage, *B. banethi* sp. nov. The latter clusters within the Western group (clade III) [2], forming a strongly supported branch in ITS1-5.8S-ITS2, *18S rRNA, cox1* trees, separately from *B. vulpes* (clade I).

Comparison of the *18S rRNA* sequences (1036 bp) revealed a high molecular identity (i.e., 99.49%) between *B. banethi* sp. nov. retrieved from #Fox1 and *Babesia* sp. MML-2014, as well as a very low homology (i.e., 88.85%) between *B. banethi* sp. nov. (#Fox1) and *B. vulpes* (#Fox2)*.* These values are consistent with previous observations among other *Babesia* species. For instance, *18S rRNA* sequences of *Babesia motasi* and *Babesia ovis* also display substantial divergence, with interspecies identities dropping below 86.6% [32]. Analogously, *18S rRNA Babesia caballi* sequences group into two main clades with intra-clade identities ranging from 95.2–100% and inter-clade identities as low as 91.5% [33].

When comparing the ITS1-5.8S-ITS2 sequences of *B. banethi* sp. nov. and *Babesia* sp. MML-2014, they were different in length (i.e., 940 bp of *B. banethi* sp. nov. vs 323 bp of *Babesia* sp. MML-2014) and displayed 98.4% molecular identity. As previously reported, an intraspecific nucleotide variation up to 3% in the ITS1–5.8S–ITS2 region, can occur among *Babesia* isolates of the same species [34]. For example, *Babesia ovata* isolates from different geographic regions share > 97% identity within the species [34]. In addition, in absence of *cox1* or *cytb* sequences for *Babesia* sp. MML-2014, which represents a limitation of the present study, the currently available nuclear markers (i.e., *18S rRNA* and ITS1-5.8S-ITS2) remain those more reliable for species delineation, supporting the hypothesis that *B. banethi* sp. nov. and *Babesia* sp. MML-2014 represent the same taxon.

On the other hand, the low overall identity (i.e., 45.15%) between ITS1-5.8S-ITS2 sequences of *B. banethi* sp. nov. and *B. vulpes,* is consistent with the divergence reported between *Babesia ovata* and *Babesia major* (i.e., 46.6%), supporting their placement as distinct *Babesia* species [32].

The above divergence across genetic markers confirms that *B. banethi* sp. nov. represents an independent evolutionary lineage rather than an intraspecific variant of *B. vulpes*. The markedly low identity observed at the ITS1-5.8S-ITS2 region, together with the substantial divergence in the *18S rRNA* gene sequences, strongly supports the genetic distinctiveness of the organism infecting #Fox1 compared with *B. vulpes* from #Fox2, suggesting that they represent separate taxa.

Considering that *cox1* sequences were not available in GenBank for *Babesia* sp. MML-2014, sequences herein obtained for *B. banethi* sp. nov. were compared with the closest related species (i.e., *Babesia negevi, B. duncani* and *B. conradae*), which displayed a nucleotide and amino acid identity ranges of 73.9–79.9% and 73.7–90.37%, respectively, such in the case of *B. vesperuginis* versus *B. conradae*, which have a nucleotide identity of 73.2% [35]. In contrast, the much lower identity values between *B. banethi* sp. nov. and *B. vulpes* (67.57% for nucleotides and 64.06% for amino acids) can be accounted for by their relevant phylogenetic distance, as they belong to distinct clades (i.e., clade III versus clade I, respectively) [2]. The phylogenetic proximity between *B. banethi* sp. nov., *B. negevi, B. duncani*, and *B. conradae* which belong to the Western group, further corroborates the above findings.

All the aforementioned phylogenetic distances between *B. banethi* sp. nov. and *B. vulpes* reflect also relevant morphological differences, being the first characterized by bigger merozoites (i.e., 1.9 × 1.2 ± 0.5 µm versus 1.33 × 0.98 μm of *B. vulpes*) and the presence of tetrads forms, which are not reported for *B. vulpes* [11]. In addition, *B. banethi* sp. nov. shares both morphological and morphometric features with *Babesia* species belonging to the Western group [26–28], having identical measures of merozoites with *B. lengau* [31], which further support the molecular closeness previously reported between these two *Babesia* species [16]. Nonetheless, while *B. duncani* and *B. conradae* are mostly confined to USA and *B. lengau* to Southern Africa [2, 11], *B. banethi* sp. nov. has been reported in different countries of the Mediterranean Basin (Israel and Italy) and from Iraq [16, 17], suggesting possible parasite distribution associated with red fox populations and their ectoparasites. Similar hypotheses have been proposed for other vector-borne pathogens of carnivores [36], highlighting the role of wildlife corridors in shaping parasite distribution also through tick vectors.

The hematological and biochemical alterations observed in #Fox1 are partially consistent with a moderate *Babesia* infection [37] as elevated globulin concentrations and reduced albumin/globulin ratio are in line with a chronic immune stimulation typically associated also with other protozoan infections, such as canine leishmaniosis [38]. The presence of rouleaux formation, leukocytosis, neutrophilia, and monocytosis further reflect a systemic inflammatory or infectious response, previously reported in infected dogs, albeit nonspecific for *Babesia* infection [37]. Conversely, the elevations in creatine phosphokinase and liver enzymes (ALT, ALP), are more likely ascribed to the traumatic event (i.e., car accident) rather than to active babesiosis, stressing the relevance of integrating parasitological findings with clinical context when evaluating wildlife clinical cases [39].

The finding of *B. banethi* sp. nov. DNA in the intestine of an engorged *I. kaiseri* tick is interpreted as ingestion of fox *B. baneti* sp. nov.-infected blood and does not suggest any replication of the parasite in the arthropod. A similar scenario was reported also for *B. vulpes*, as its DNA was detected in several tick species collected from wild and domestic canids (i.e., *Ixodes hexagonus*, *Ixodes ricinus*, *Ixodes canisuga* and *Rhipicephalus sanguineus*), yet their vectorial competence has never been demonstrated [11]. Therefore, future studies focusing on the breeding of ticks collected from *Babesia* spp. infected fox or on experimental transmission are needed to clarify the life cycle of both *B. banethi* sp. nov. and *B. vulpes*. Specifically, knowledge about tick vectors of these *Babesia* species, as well as on alternative transmission routes (e.g., fighting, predation between foxes) may be relevant for understanding the health risk of dogs, as already reported for *B. vulpes* [14]. Indeed, foxes are synanthropic animals largely occurring in the Mediterranean basin, where they share endo- and ecto-parasites with domestic carnivores [36].

## Conclusions

This study describes a new *Babesia* sp., different from *B. vulpes*, infecting red foxes. Specifically, data presented provide morphological description, differential diagnosis and genetic characterization of *B. banethi* sp. nov., a new taxon within the *Babesia* s.l. complex (Western group). Given the synanthropic occurrence of foxes and domestic dogs in the Mediterranean regions, future studies should elucidate the transmission dynamics of *B. banethi* sp. nov., its host range and geographical distribution.

## Supplementary Information

Additional file 1. Fig. S1. Multiple sequence alignment of the 5.8S-ITS2 nucleotide sequences of *Babesia banethi *sp. nov. from #Fox1 (Italy), *Babesia *sp. MML-2014 (GenBank accession no. KR709304.1) and reference sequences belonging to the same clade (i.e., Western group, Clade III). The alignment was generated and visualized using Jalview v2.11.5.0.

Additional file 2. Fig. S2. Multiple sequence alignment of the *cox*1 nucleotide sequences of *Babesia banethi* sp. nov. from #Fox1 (Italy) and reference sequences belonging to the same clade (i.e., Western group, Clade III). The alignment was generated and visualized using Jalview v2.11.5.0.

Additional file 3. Fig. S3. Multiple sequence alignment of the *cox*1 amino acid sequences of *Babesia banethi *sp. nov. from #Fox1 (Italy) and reference sequences belonging to the same clade (i.e., Western group, Clade III). The blue arrow indicates positions conserved within the Western group but distinct in *B. banethi* sp. nov. The alignment was generated and visualized using Jalview v2.11.5.0.

## Data Availability

All data supporting the main conclusions of this study are included in the manuscript.

## Abbreviations

DNA: Deoxyribonucleic acid
PCR: Polymerase chain reaction
cPCR: Conventional polymerase chain reaction
qPCR: Quantitative (real-time) polymerase chain reaction
ICZN: International Code of Zoological Nomenclature
rRNA: Ribosomal ribonucleic acid
ITS: Internal transcribed space
CBC: Complete blood cell count
*cox*1: Cytochrome oxidase subunit 1
*cytB*: Cytochrome *b*
BLAST: Basic Local Alignment Search Tool
NCBI: National Center for Biotechnology Information
mtDNA: Mitochondrial deoxyribonucleic acid
MSA: Multiple sequence alignment
CPK: Creatine phosphokinase
AST: Aspartate aminotransferase
ALT: Alanine aminotransferase
