# Supplementary figures and images for: Babesia banethi sp. nov. in red foxes
Source: Parasit Vectors. 2025 Dec 11;19:3. doi: 10.1186/s13071-025-07179-y (PMC12765302; doi:10.1186/s13071-025-07179-y)

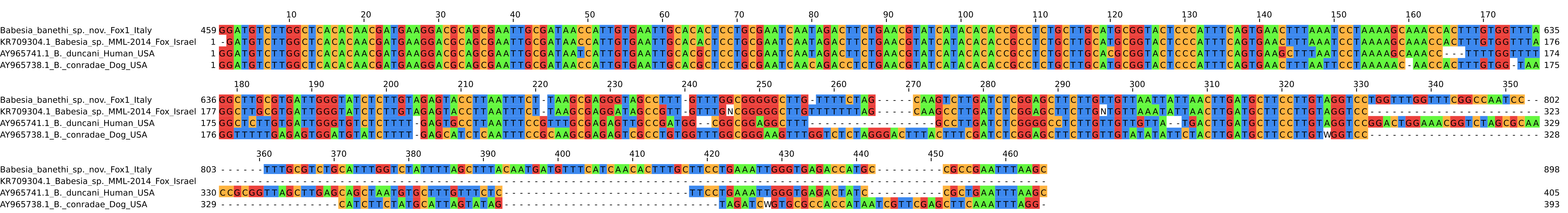

Supplement: Supplementary file 1 — Additional file 1. Fig. S1. Multiple sequence alignment of the 5.8S-ITS2 nucleotide sequences of Babesia banethi sp. nov. from #Fox1 (Italy), Babesia sp. MML-2014 (GenBank accession no. KR709304.1) and reference sequences belonging to the same clade (i.e., Western group, Clade III). The alignment was generated and visualized using Jalview v2.11.5.0. [file 13071_2025_7179_MOESM1_ESM.tiff]

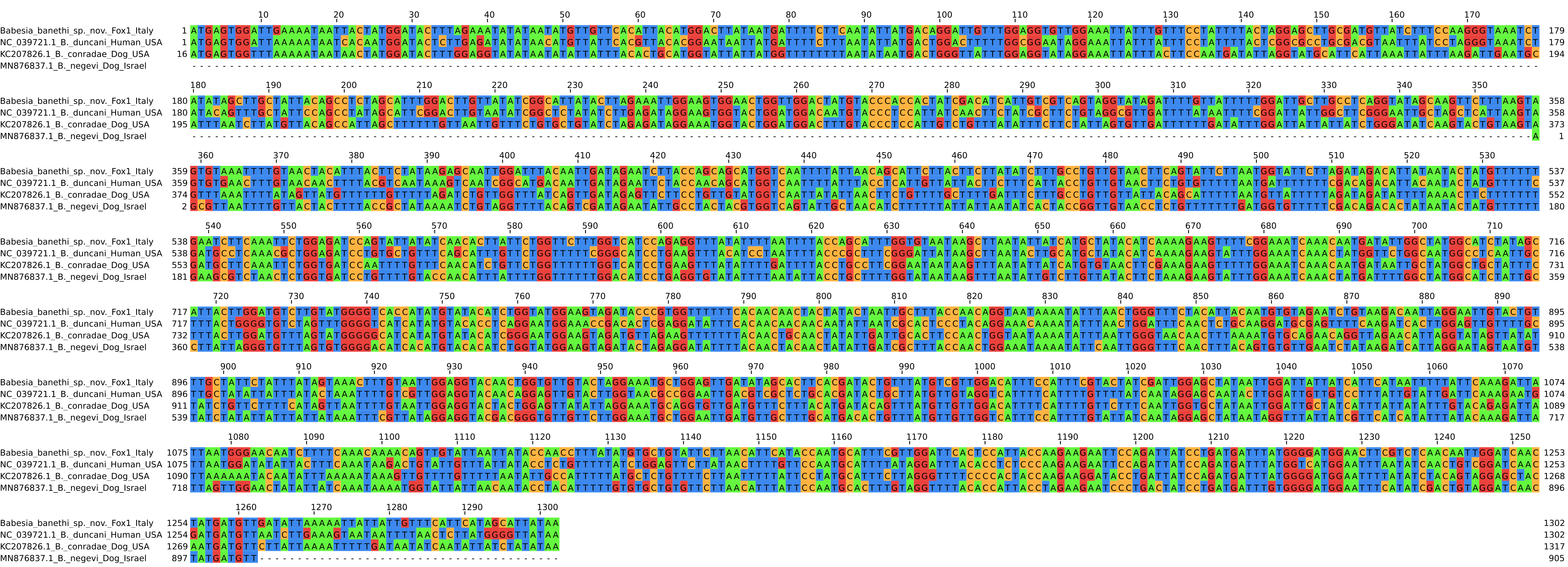

Supplement: Supplementary file 2 — Additional file 2. Fig. S2. Multiple sequence alignment of the cox1 nucleotide sequences of Babesia banethi sp. nov. from #Fox1 (Italy) and reference sequences belonging to the same clade (i.e., Western group, Clade III). The alignment was generated and visualized using Jalview v2.11.5.0. [file 13071_2025_7179_MOESM2_ESM.tiff]

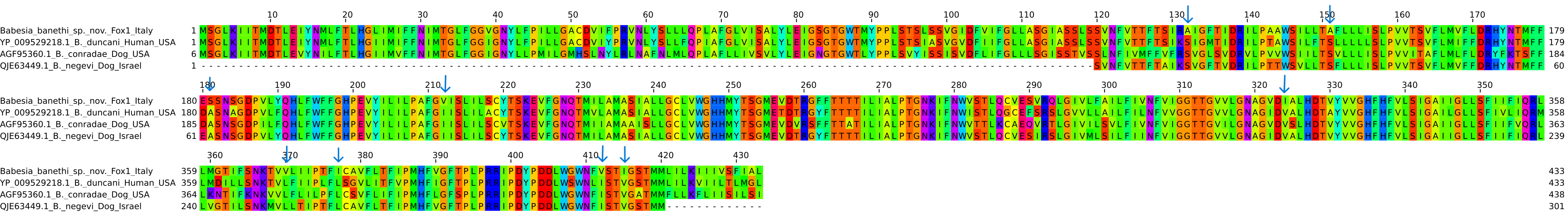

Supplement: Supplementary file 3 — Additional file 3. Fig. S3. Multiple sequence alignment of the cox1 amino acid sequences of Babesia banethi sp. nov. from #Fox1 (Italy) and reference sequences belonging to the same clade (i.e., Western group, Clade III). The blue arrow indicates positions conserved within the Western group but distinct in B. banethi sp. nov. The alignment was generated and visualized using Jalview v2.11.5.0. [file 13071_2025_7179_MOESM3_ESM.tiff]
